# Incidence, Outcomes and Risk Factors for Atrial Fibrillation in Patients With *JAK2^V617F^*‐Positive Myeloproliferative Neoplasms

**DOI:** 10.1002/cam4.71015

**Published:** 2025-07-03

**Authors:** Guangshuai Teng, Ke Shang, Yuhui Zhang, Yifan Duan, Chenxiao Du, Yan Wang, Yanqi Li, Huiqin Zhang, Lan Peng, Xiaojing Wei, Gary Tse, Yuan Zhou, Gregory Y. H. Lip, Tong Liu, Wei Yang, Minghui Duan, Jie Bai

**Affiliations:** ^1^ Department of Hematology The Second Hospital of Tianjin Medical University Tianjin China; ^2^ Department of Cardiology The Second Hospital of Tianjin Medical University Tianjin China; ^3^ Tianjin Key Laboratory of Ionic‐Molecular Function of Cardiovascular Disease Tianjin China; ^4^ Tianjin Institute of Cardiology Tianjin China; ^5^ School of Nursing and Health Studies Hong Kong Metropolitan University Hong Kong China; ^6^ State Key Laboratory of Experimental Hematology, National Clinical Research Center for Blood Diseases, Haihe Laboratory of Cell Ecosystem, Institute of Hematology & Blood Diseases Hospital Chinese Academy of Medical Sciences & Peking Union Medical College Tianjin China; ^7^ Tianjin Institutes of Health Science Tianjin China; ^8^ Liverpool Centre for Cardiovascular Science at University of Liverpool Liverpool John Moores University and Liverpool Heart & Chest Hospital Liverpool UK; ^9^ Danish Center for Health Services Research, Department of Clinical Medicine Aalborg University Aalborg Denmark; ^10^ Department of Hemotology Shengjing Hospital of China Medical University Liaoning China; ^11^ Department of Hematology, Peking Union Medical College Hospital Chinese Academy of Medical Sciences & Peking Union Medical College Beijing China

**Keywords:** atrial fibrillation, cytokines, gene mutation, *JAK2* ^*V617F*^, myeloproliferative neoplasms

## Abstract

**Background:**

The incidence, outcomes and risk factors for AF in the *JAK2* ^*V617F*^‐positive MPN patients are still unknown.

**Methods:**

The clinical profiles of patients with *JAK2* ^*V617F*^‐positive MPN were retrospectively analyzed. Multivariable Cox regression analysis was performed to identify risk factors of AF, thereby developing a risk prediction model.

**Results:**

A total of 439 patients were included (age 57 [12–87] years; 51.3% male). AF was associated with higher risks of stroke (*p* = 0.036, HR = 1.987, 95% CI 1.047–3.772) and mortality (*p* < 0.001, HR = 3.857, 95% CI 1.836–8.103). Multivariable Cox regression showed that *TET2* mutation (*p* = 0.042, HR = 4.361, 95% CI 1.053–18.056) and increased IL‐1β (*p* = 0.012, HR = 5.476, 95% CI 1.547–28.123) were significant risk factors for AF in patients with *JAK2* ^*V617F*^‐positive MPN. Nomograms were constructed, allowing patients to be categorized into high‐ and low‐risk groups. The 10‐year AF‐free survival rate was significantly lower in the high‐risk group (62% vs. 91.7%; log‐rank test: *p* = 0.002). The validation cohort confirmed that the survival without AF in the high‐risk group was significantly worse than that in the low‐risk group. The use of either interferon‐α or ruxolitinib, was associated with longer AF‐free survival in the high‐risk group (*p* < 0.05).

**Conclusion:**

AF was significantly associated with higher risks of stroke and mortality. *TET2* mutation and increased IL‐1β were independent risk factors of AF in patients with *JAK2* ^*V617F*^‐positive MPN.

## 1 Introduction

Clonal hematopoiesis of indeterminate potential (CHIP) is an age‐associated phenomenon, resulting from somatic mutations in hematopoietic stem cells that generate expansion of clones harboring mutations. *DNMT3A*, *TET2*, *ASXL1*, and *JAK2* are the commonest genes implicated in CHIP [1]. They are commonly found in hematologic myeloid neoplasms, with *JAK2* ^*V617F*^ as the most common site for *JAK2* mutation, which is the most common driver mutation for myeloproliferative neoplasm (MPN) and is present in 95% of patients with polycythemia vera (PV), and 50%–60% of patients with essential thrombocythemia (ET) and primary myelofibrosis (PMF) [2].

Significant associations between *JAK2* and the occurrence of atrial fibrillation (AF) are observed in the healthy population [1]. Moreover, the incidence of AF in patients with MPN is higher than the general population [3, 4]. AF is the most common arrhythmia observed in clinical practice, with a similar age of onset to MPN [4], and associated with elevated risks of thromboembolism and death [5, 6, 7, 8]. While significant progress has also been made in the management of AF, the specific risk factors for AF in the MPN patient population have not been fully elucidated.

*JAK2* ^*V617F*^ mutation leads to Janus kinase activation and causes inflammation, contributing to the development of cardiovascular and cerebrovascular events in MPN [9]. Higher *JAK2* ^*V617F*^ burden contributes to higher levels of proinflammatory factors and higher risks of cardiovascular events [2, 6]. In addition, epigenetic abnormalities such as inflammatory dysregulation and *TET2* deletion can also enhances risk for AF [10]. Thus, genomic instability and inflammatory dysregulation are common mechanisms in the development of MPN and AF [11]. However, there are few reports on whether inflammation and genetic landscape are related to the occurrence of AF in MPN patients.

In this study, we analyzed the occurrence, clinical profiles, cytokine levels, gene mutation characteristics, and outcomes of AF in 241 patients with *JAK2* ^*V617F*^‐positive MPN, and second, explored the risk factors and drug therapies associated with the occurrence of AF in patients with *JAK2* ^*V617F*^‐positive MPN.

## 2 Methods

### 2.1 Study Population

This study was approved by the Ethics Committees of the Second Hospital of Tianjin Medical University, Peking Union Medical College Hospital, Chinese Academy of Medical Sciences & Peking Union Medical College, and Shengjing Hospital of China Medical University, and was conducted in accordance with the Declaration of Helsinki. Owing to the retrospective nature of this study with no intervention or additional contact with the patients beyond routine clinical care, the need for informed consent was waived by the committees.

This was a retrospective analysis of patients with *JAK2* ^*V617F*^‐positive MPN who attended the Department of Hematology of the Second Hospital of Tianjin Medical University, the Department of Hematology, Peking Union Medical College Hospital, Chinese Academy of Medical Sciences & Peking Union Medical College, and the Department of Hematology of Shengjing Hospital of China Medical University, from November 2017 to March 2025. Diagnosis of MPN was based on the 2022 diagnostic classification criteria from World Health Organization (WHO) [12] and clinical and laboratory data were confirmed by ≥ 3 clinicians and bone marrow biopsies were reassessed by three pathologists before patients were included in the study.

The diagnosis of AF was determined by codes in the International Classification of Diseases and text searches in patients' electronic medical records and verified by electrocardiogram (ECG) data or Holter monitors, with AF associated with surgery, valvular disease, or cardiomyopathy excluded. Information on all patients was confirmed by means of outpatient follow‐up and telephone follow‐up.

Detailed methods are provided in the Supporting Information S1 and S2.

### 2.2 Statistical Analysis

Patients were divided into AF group (AF) and no AF group (non‐AF) according to whether they developed AF; arterial thrombosis was defined as stroke (stroke was defined as any ischemic event with rapid onset of a focal or global neurologic deficit or other neurologic signs/symptoms consistent with stroke), acute coronary syndrome (ACS), peripheral arterial thrombosis, and other arterial thrombosis. Venous thrombosis was defined as pulmonary embolism, visceral venous thrombosis, deep vein thrombosis of extremities, and other venous thrombosis.

Paroxysmal AF was defined as recurrent, electrocardiographically documented AF episodes or single electrocardiographically documented AF episode together with the medical history, which shows spontaneous termination within 7 days after onset. Persistent AF was defined as AF with cardioversion performed or planned, or AF that lasts more than 7 days and does not meet the criteria for permanent AF. Permanent AF was defined as AF at the last ECG plus the presence of arrhythmia as diagnosed by a physician [13].

AF‐free survival was defined as the time from the diagnosis of MPN to the occurrence of AF or the last follow‐up. The incidence of events during follow‐up was calculated in 1000 person‐years. Statistical analysis was performed using SPSS 24.0, and non‐normally distributed data were described as median (range). Qualitative data were compared using the chi‐square test and Fisher's test, and groups were compared using the Mann–Whitney *U* test and Kruskal–Wallis test. Spearman's correlation coefficients were used to assess correlations between gene variant allele frequency (VAF) and the level of cytokines. The Kaplan–Meier method was used for survival analysis. Multivariable Cox regression was performed. Variables were selected based on *p* < 0.05, on multivariable regression.

Variables were then used to construct a nomogram for predicting 2‐, 5‐, and 10‐year AF‐free survival rates. The discriminant ability and accuracy of the model were assessed using calibration curves constructed by the “rms” and “survival” packages of the R software [14]. The time receiver operating characteristic (tROC) curve was employed to determine the optimal cut‐off value for the prognostic index. A validation cohort of MPN patients who attended the Department of Hematology of the Second Hospital of Tianjin Medical University was used to validate the model. *p* < 0.05 was considered statistically significant.

## 3 Results

### 3.1 AF in *JAK2* ^*V617F*^ ‐Positive MPN Patients

Initially 454 patients with *JAK2* ^*V617F*^‐positive MPN were identified but 15 (3.3%) patients were lost to follow‐up. Among the remaining 439 patients, AF, heart failure, renal insufficiency, progression to acute myeloid leukemia (AML), thromboembolism, hemorrhage and death occurred in 29 (6.6%), 47 (19.3%), 18 (7.8%), 5 (1.1%), 120 (33.5%), 22 (9.1%) and 40 (9.1%) patients, respectively (Table S1).

During the follow‐up period, 29 patients (6.6%) developed AF: 15 cases (51.7%) of paroxysmal AF, 10 cases (34.5%) of persistent AF, and 4 cases (13.8%) of permanent AF (Figure S1A). The proportion of AF in PV, ET, Post PV/ET MF, and PMF was 31% (9/29), 20.7% (6/29), 24.1% (7/29), and 24.1% (7/29), respectively (Figure S1B). The incidence rate of AF was 9.2/1000 (95% CI [6.3/1000, 13.4/1000]) person‐years. The 1‐, 5‐, 10‐, and 15‐year rates of AF were 2.8%, 5.4%, 8.3%, and 12.6%, respectively (Figure S1C).

### 3.2 Clinical Profiles

Patients were divided into AF subgroup (*n* = 29) and no‐AF group (*n* = 410) based on the occurrence of AF events. Compared with the non‐AF group, the AF subgroup had a higher age of onset, absolute monocyte count, left atrial diameter, proportion of patients with reticular fibers ≥ grade 2, splenomegaly, *V617F*% > 50%, comorbidities, and mortality, while hemoglobin levels and LVEF were lower (*p* < 0.05) (Table 1).

**TABLE 1:** Characteristics of AF in *JAK2* ^*V617F*^‐positive MPN patients.

	AF group (*n* = 29)	Non‐AF group (*n* = 410)	*p*
Follow‐up time, years media (range)	5 (1–21)	6 (1–29)	0.110
Age, media (range)	68 (38–85)	56 (12–87)	< 0.001***
Age > 60, *n* (%)	20 (69%)	161 (39.3%)	0.003**
Male, *n* (%)	15 (51.7%)	210 (51.2%)	1.000
Diagnosis			
PV, *n* (%)	9 (31.0%)	295 (72.0%)	< 0.001***
ET, *n* (%)	6 (20.7%)	35 (8.5%)	
PostPV/ET MF, *n* (%)	7 (24.1%)	58 (14.1%)	
PMF, *n* (%)	7 (24.1%)	22 (5.4%)	
At the time of diagnosis			
HB, g/L media (range)	150 (60–237)	170 (33–261)	0.002**
WBC, ×10^9^/L media (range)	10.42 (4.20–32.28)	9.78 (1.34–41.07)	0.958
Monocytes, ×10^9^/L media (range)	0.62 (0.39–4.92)	0.43 (0.11–3.53)	< 0.001***
Monocytes > 0.6 × 10^9^/L, *n* (%)	11 (47.8%)	49 (17.6%)	0.002**
PLT, ×10^9^/L media (range)	503 (34–1658)	465 (41–1749)	0.295
Abnormal karyotype, *n* (%)	2 (8.0%)	22 (10.1%)	1.000
Reticular fiber ≥ grade 2, *n* (%)	14 (48.3%)	80 (23.5%)	0.006**
Splenomegaly, *n* (%)	20 (69.0%)	162 (39.8%)	0.003**
*V617F*% > 50%, *n* (%)	17 (63.0%)	138 (38.0%)	0.014*
Hypertension, *n* (%)	6 (22.2%)	71 (24.0%)	1.000
Hyperlipidemia, *n* (%)	7 (25.9%)	60 (23.9%)	0.815
Diabetes, *n* (%)	2 (7.7%)	15 (5.2%)	0.639
Smoking, *n* (%)	3 (10.7%)	40 (11.5%)	1.000
LVEF, % media (range)	60.5 (46–68)	64 (52–74)	0.001**
Left atrial diameter, mm media (range)	39.65 (33.4–51.5)	37.1 (24.8–52.7)	0.032*
LVEDD, mm media (range)	47.75 (41.7–60)	47.3 (38.7–63.5)	0.677
BMI, kg/m^2^ media (range)	24.73 (17.72–28.34)	23.24 (16–34.2)	0.167
Serum uric acid, μmol/L media (range)	399.6 (169.8–855.8)	354.4 (141.8–759.6)	0.157
Hyperuricemia, *n* (%)	13 (52.0%)	93 (35.4%)	0.128
Therapy			
Hydroxyurea, *n* (%)	17 (60.7%)	132 (41.4%)	0.071
Interferon‐α, *n* (%)	18 (62.1%)	297 (77.1%)	0.074
Ruxolitinib, *n* (%)	3 (10.3%)	88 (26.7%)	0.072
Antiplatelet, *n* (%)	16 (57.1%)	201 (67.9%)	0.294
Anticoagulant, *n* (%)	2 (8.0%)	10 (4.6%)	0.365
Hemorrhage, *n* (%)	4 (17.4%)	18 (8.3%)	0.242
Thrombosis, *n* (%)	16 (59.3%)	104 (31.4%)	0.005**
Arterial thrombosis, *n* (%)	17 (63.0%)	94 (28.4%)	< 0.001***
Venous thrombosis, *n* (%)	1 (3.7%)	26 (7.9%)	0.708
ACS	6 (24.0%)	16 (7.3%)	0.015*
Stroke	16 (64%)	54 (24.8%)	< 0.001***
Heart failure, *n* (%)	16 (64%)	31 (14.2%)	< 0.001***
Renal insufficiency, *n* (%)	6 (24.0%)	12 (5.8%)	0.007**
Death, *n* (%)	14 (48.3%)	26 (6.3%)	< 0.001***

Abbreviations: ACS, acute coronary syndrome; BMI, body mass index; ET, essential thrombocythemia; HB, hemoglobin; LVEDD, left ventricular end‐diastolic diameter; LVEF, left ventricular ejection fraction; MF, myelofibrosis; PLT, platelet count; PMF, primary myelofibrosis; PV, polycythemia vera; WBC, white blood cell.

* *p* < 0.05.

** *p* < 0.01.

*** *p* < 0.001.

### 3.3 Gene Mutation Characteristics and Cytokine Levels

In this study, 375 patients underwent next generation gene sequencing at the time of diagnosis. Common concomitant gene mutations included *DNMT3A* mutation, *TET2* mutation, *ASXL1* mutation, *FAT1* mutation, *EP300* mutation, etc., of which the proportion of patients with *TET2* mutation in the AF group was significantly higher when compared with the no‐AF group (*p* < 0.05) (Table S2).

**FIGURE 1:**
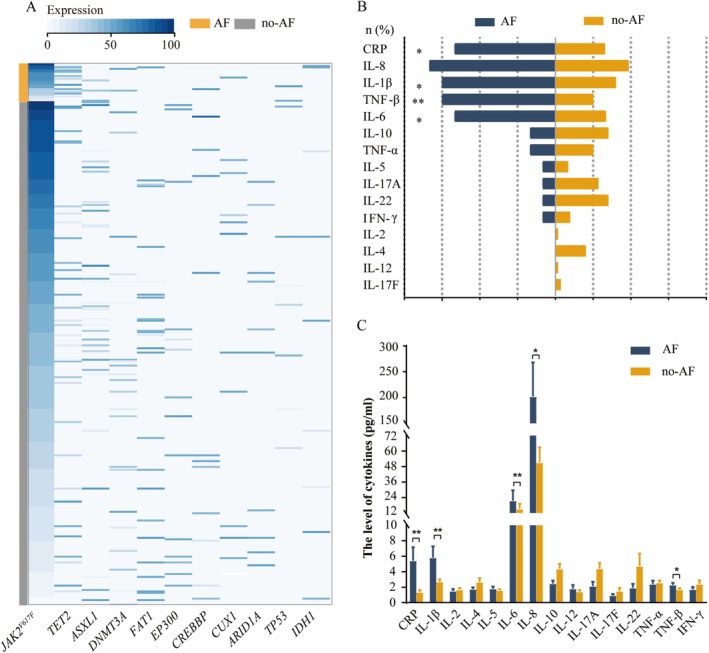
The gene mutation spectrum and the cytokines in *JAK2* ^*V617F*^‐positive MPN patients. (A) The gene mutation spectrum of 375 *JAK2* ^*V617F*^‐positive MPN patients. (B) Comparison of the proportion of the elevated rate of cytokines in *JAK2* ^*V617F*^‐positive MPN patients between AF group and no‐AF group. (C) Comparison of the levels of cytokines in *JAK2* ^*V617F*^‐positive MPN patients between AF group and no‐AF group. AF, atrial fibrillation; CRP, C‐reactive protein; IFN, interferon; IL, interleukin; TNF, tumor necrosis factor. *p < 0.05; **p < 0.01.

The results comparing the mutation load between the two groups showed that the VAF of *TET2* was significantly higher in the AF group (*p* < 0.05), with a trend towards an increase in *V617F*%, whereas no significant differences were observed in the VAFs of *ASXL1*, *DNMT3A*, *FAT1*, and *EP300* between the two groups (Figure 1A, Table S3).

Plasma cytokines and CRP were quantified in 90 patients and 140 patients respectively. The proportion of patients with CRP, interleukin (IL)‐1β, IL‐6 or tumor necrosis factor (TNF)‐β above the upper limit of normal in AF group was significantly higher than those in no‐AF group (*p* < 0.05). The levels of CRP, IL‐1β, IL‐6, IL‐8, and TNF‐β in AF group were also higher than those in no‐AF group (*p* < 0.05) (Figure 1B,C, Tables S2 and S3).

**FIGURE 2:**
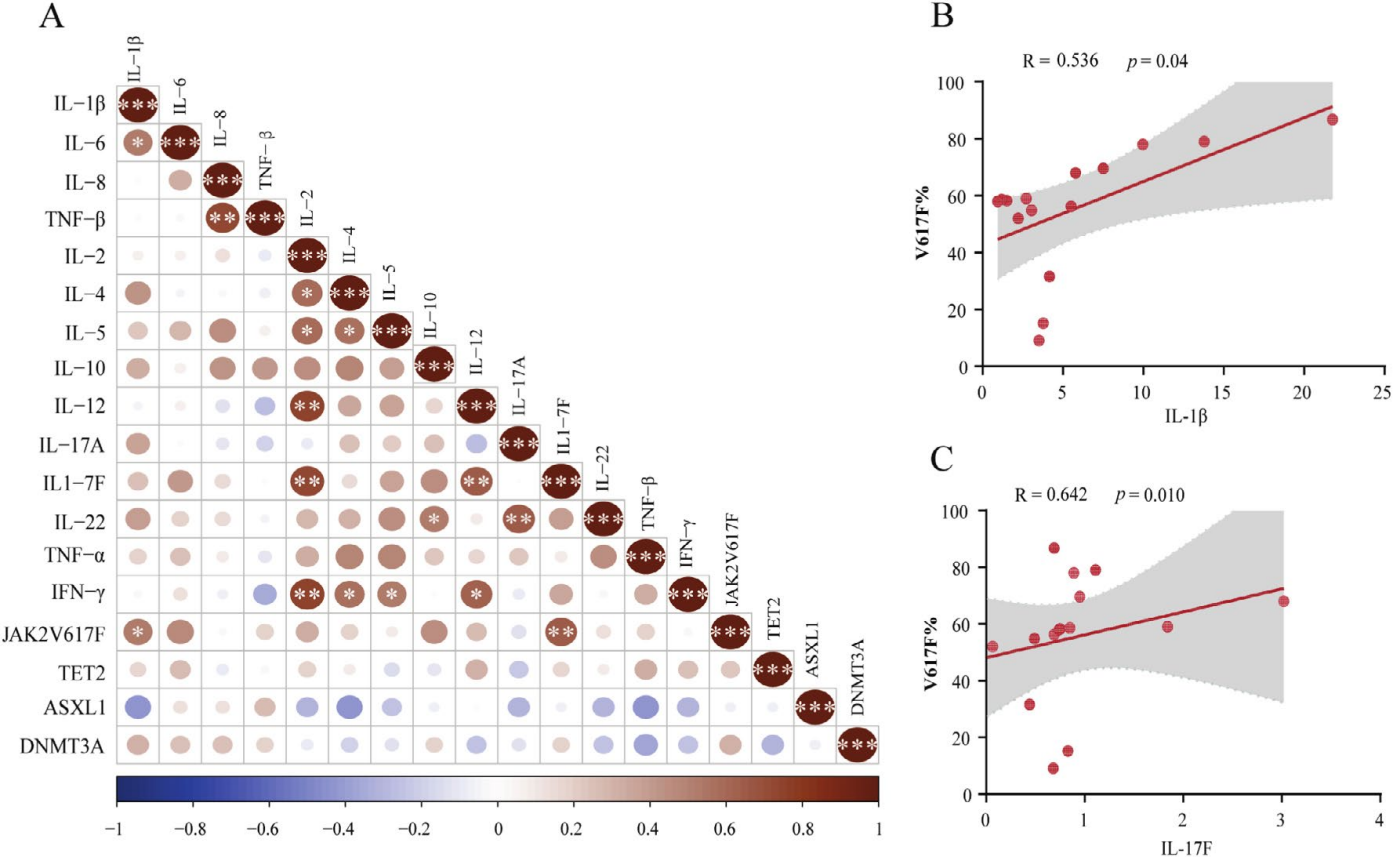
Correlation analysis between gene mutations and levels of cytokines in the AF group. (A) Correlation analysis between *JAK2* ^*V617F*^, *TET2*, *ASXL1*, *DNMT3A*, and cytokines. (B) Correlation between *V617F*% and IL‐1β. (C) Correlation between *V617F*% and IL‐17F. CRP, C‐reactive protein; IFN, interferon; IL, interleukin; TNF, tumor necrosis factor. *p < 0.05; **p < 0.01; ***p < 0.001.

Correlation analysis between gene mutation load and cytokine levels was performed. A significant positive correlation between *V617F*% and IL‐1β level or IL‐17F level was found in the AF group (*p* = 0.04 and *p* = 0.01). However, no significant correlation was observed between *V617F*% and other cytokines in the AF group, nor between the VAFs of *TET2*, *DNMT3A*, and *ASXL1* and cytokines (*p* > 0.05) (Figure 2A–C).

### 3.4 Clinical Outcomes

In the AF group, 4 cases (17.4%) of hemorrhage, 14 cases (48.3%) of death, and 16 cases (59.3%) of thromboembolism occurred, including 17 cases (63%) of arterial thrombosis, 1 case (3.7%) of venous thrombosis, 6 cases (24.0%) of ACS, and 16 cases (64%) of stroke (Table 1).

AF was associated with the higher mortality and higher incidence of thrombosis, arterial thrombosis, ACS, and stroke in patients with *JAK2* ^*V617F*^‐positive MPN (*p* < 0.05). AF was associated with higher risks of stroke (*p* = 0.036, HR = 1.987, 95% CI 1.047–3.772) and mortality (*p* < 0.001, HR = 3.857, 95% CI 1.836–8.103) despite adjustments to gender, age, and BMI (Table 2).

**TABLE 2:** Impact of AF on the clinical outcomes in patients with *JAK2* ^*V617F*^‐positive MPN.

Clinical outcome	Univariable analysis		Multivariable analysis		
	*χ* ^2^	*p*	HR	95% CI	*p*
Death	62.539	< 0.001***	3.857	1.836–8.103	< 0.001***
Thrombosis	12.390	< 0.001***	1.417	0.775–2.594	0.258
Arterial thrombosis	17.487	< 0.001***	1.577	0.874–2.846	0.131
Venous thrombosis	0.165	0.684	1.185	0.150–9.362	0.872
ACS	8.801	0.003**	2.833	0.883–9.096	0.080
Stroke	10.340	0.001**	1.987	1.047–3.772	0.036*

*Note:* Multivariate analysis adjusting for covariates such as gender, age, and BMI.

Abbreviations: ACS, acute coronary syndrome; BMI, body mass index.

* *p* < 0.05.

** *p* < 0.01.

*** *p* < 0.001.

### 3.5 Risk Factors for AF Development

**FIGURE 3:**
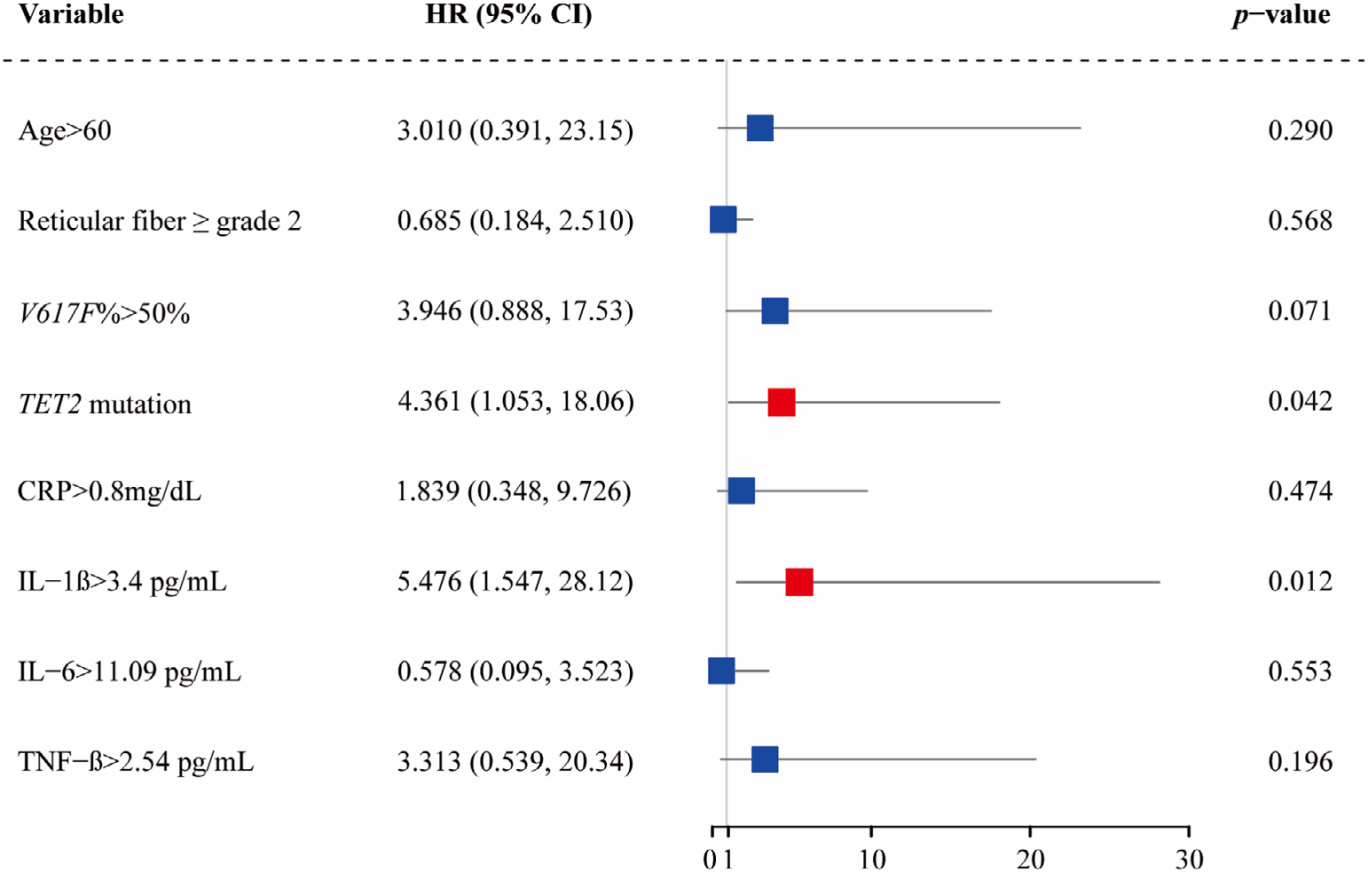
Risk factors for AF‐free survival in *JAK2* ^*V617F*^‐positive myeloproliferative neoplasm patients. CRP, C‐reactive protein; IL, interleukin; TNF, tumor necrosis factor.

Univariable Cox regression was performed for clinical and laboratory data. Multivariable regression was performed for variables achieving *p* < 0.05 on the univariate analysis: age > 60 years, reticular fiber ≥ grade 2, *V617F*% > 50%, *TET2* mutations, CRP > 0.8 mg/dL, IL‐1β > 3.4 pg/mL, IL‐6 > 11.09 pg/mL, TNF‐β > 2.54 pg/mL. *TET2* mutations (*p* = 0.042, HR = 4.361, 95% CI [1.053–18.056]) and increased IL‐1β (*p* = 0.012, HR = 5.476, 95% CI [1.547–28.123]) were significant risk factors associated with AF (Figure 3, Table S4).

**FIGURE 4:**
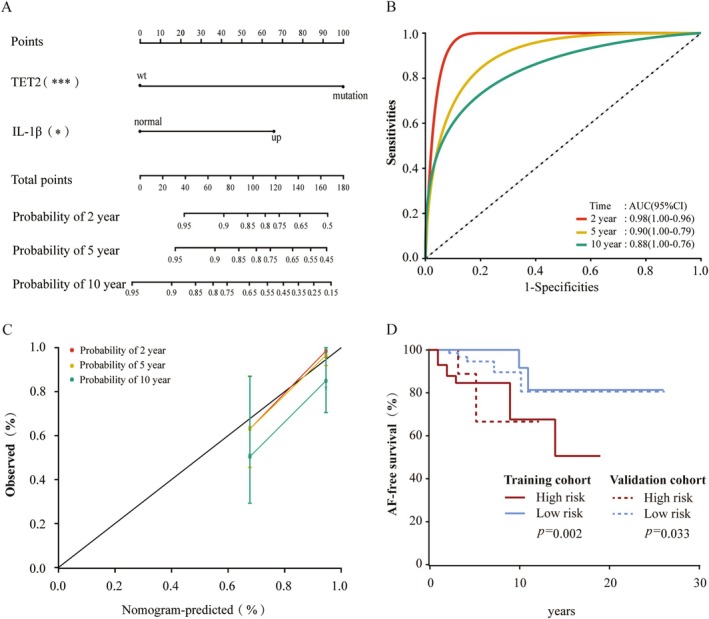
Construction of a prediction nomogram for AF prediction. (A) Establishment of AF‐free survival nomograms. (B) Receiver operating characteristic curves were produced at 2‐, 5‐, and 10‐year. (C) The calibration curves for predicting AF‐free survival at 2‐, 5‐, and 10‐year. (D) Kaplan–Meier survival curves showing AF‐free survival according to prognostic index in the training cohort (solid line, *n* = 89) and in the validation cohort (dashed line, *n* = 112). IL, interleukin. *p < 0.05; ***p < 0.001.

A nomogram (Figure 4A) was constructed based on multivariable Cox regression analysis to predict the probability of AF at 2, 5, and 10 years. ROC analysis showed that the area under the curve (AUC) values for 2, 5, and 10 years of follow‐up were 0.98, 0.90, and 0.88, respectively (Figure 4B). The calibration curve results demonstrated that the nomogram robustly predicted AF‐free survival (Figure 4C). The optimal cutoff prognostic index was −1.00, based on which patients were classified into high‐risk and low‐risk groups. Kaplan–Meier survival analysis revealed that the 10‐year AF‐free survival rate in the high‐risk group was significantly lower than that in the low‐risk group (62% vs. 91.7%, *p* = 0.002; Figure 4D, solid line). To further validate the stability of the model, a Kaplan–Meier survival analysis was conducted in an MPN validation cohort, which showed that the 10‐year AF‐free survival rate in the high‐risk group was significantly lower than in the low‐risk group (62.5% vs. 80.7%, *p* = 0.033) (Figure 4D, dashed line).

### 3.6 Impact of MPN Treatment

**FIGURE 5:**
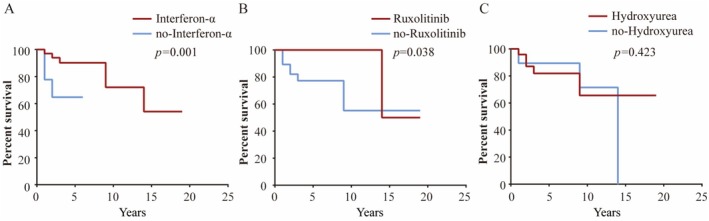
Kaplan–Meier survival curves showing AF‐free survival according to treatment status in the high‐risk group; (A) Interferon‐α, (B) ruxolitinib, and (C) hydroxyurea.

The use of interferon‐α or ruxolitinib was associated with longer AF‐free survival in the high‐risk group (*p* < 0.05) (Figure 5A,B). In contrast, hydroxyurea had no significant effect on the AF‐free survival of patients in the high‐risk group (Figure 5C). In the low‐risk group, treatment status had no significant impact on AF‐free survival (Figure S2).

## 4 Discussion

To the best of our knowledge, our study is the first to explore a risk prediction model for AF in patients with MPN using clinical parameters, inflammatory biomarkers, and gene mutations by next‐generation sequencing. Increased IL‐1β and *TET2* mutation were independent risk factors for AF.

The main findings of this study on a cohort of patients with *JAK2* ^*V617F*^‐positive MPN are as follows: (i) the incidence rate of AF was 9.2/1000 (95% CI [6.3/1000, 13.4/1000]) person‐years; (ii) those with AF were older and had higher absolute monocyte count, larger left atrial diameter, heavier *V617F*% load, and lower hemoglobin levels and left ventricular ejection fraction; (iii) the risks of stroke and death were 1.987 and 3.857 fold greater respectively in patients with AF; (iv) increased IL‐1β and *TET2* mutation were independent risk factors for AF; and (v) interferon‐α or ruxolitinib, but not hydroxyurea, was associated with AF‐free survival in the high‐risk group. Exploring a risk prediction model with ROC analysis showed that the AUC values for 2, 5, and 10 years of follow‐up were 0.98, 0.90, and 0.88, respectively.

Cancer and other types of malignancies are established risk factors of AF [15]. In patients with MPN, the incidence of AF was 8.7%–18.7% [3, 4], while the incidence of AF in MPN patients younger than 60 years was about 5.1% [4], which is higher than that in the general population. The results of this study were similar to those reported in the Chinese cohort study [3], but slightly lower than the incidence of AF in patients with *JAK2* ^*V617F*^‐positive MPN reported in a French cohort study (19.7%) [4].

The incidence of thrombosis in patients with *JAK2* ^*V617F*^‐positive MPN was as high as 43.9% [2], of which the incidence of stroke was significantly higher than other types of embolism [2, 5]. *JAK2* ^*V617F*^ mutation, age and hypertriglyceridemia were important risk factors for stroke events in patients with MPN [16]. Factors such as uncoordinated myocyte activity due to impaired atrial contractility caused by AF and atherosclerosis of aortic arch can increase the risk of blood stasis and thromboembolism and are also closely related to the occurrence of stroke [17].

Our study found that AF was significantly associated with higher risks of stroke in patients with *JAK2* ^*V617F*^‐positive MPN after adjusting for confounding factors such as gender and age. According to other studies, advanced age, hypertension, and other clinical factors were important AF risk predictors [18]. These variables were also included in this study, but their statistical significance was lost on multivariable regression. Such differences may also be due to the fact that the patients with *JAK2* ^*V617F*^‐positive MPN were studied, and there were more patients with *TET2* mutation. Both *JAK2* ^*V617F*^ mutation and *TET2* mutation can cause increased secretion of proinflammatory factors in the body. Specific factors such as MPN disease itself and gene mutation are the main reasons for mediating the inflammatory response and promoting the occurrence of AF, playing more important roles than clinical factors. In addition, clinical risk factors, inflammatory, and genetic findings may show complex or latent interactions to mediate AF risk, which is beyond the scope of this study.

Previous studies have shown that inflammatory cytokines such as IL‐1, IL‐6, and IL‐8 were elevated in patients with MPN [19, 20, 21]. Among them, IL‐1β is one of the important members of the IL‐1 cytokine family and is a major regulator of inflammation. It is secreted by monocytes and macrophages and depends on inflammasomes to become immunocompetent and induce immune responses and induce its own expression through a positive feedback loop in autocrine or paracrine mechanisms [22, 23]. IL‐1β promotes the development of AF by shortening the atrial effective refractory period, promoting atrial fibrosis [24] and exacerbating atrial electrical remodeling [25]. *JAK2* ^*V617F*^ mutation is the most common driver mutation for MPN and is also the mutation that is most closely related to cardiovascular risk and thrombosis.

In case of *JAK2* ^*V617F*^ mutation, IL‐1β secretion is significantly increased compared with normal controls, involving in thrombosis [26, 27]. *TET2* mutation is the common concomitant gene mutation for MPN. *TET2* deletion promotes the occurrence of cardiovascular disorders such as cardiac failure and atherosclerosis by the activation of IL‐1β/NLRP3 inflammasome through macrophages [28, 29]. In a *TET2* mouse model, NLRP3 inflammasome activation led to higher levels of IL‐1β, resulting in abnormal calcium regulation and increased AF susceptibility [10]. These experimental findings strongly support and provides a biological basis for the relevance of IL‐1β, *TET2* mutation and AF for our patients with *JAK2* ^*V617F*^‐positive MPN.

In this study, we found a positive correlation between IL‐1β levels and *V617F*% in AF patients, consistent with previous findings [21]. We speculated that, in addition to *TET2* deletion, *JAK2* ^*V617F*^ mutation itself was also an important factor inducing increased IL‐1β expression in patients with MPN, and increased IL‐1β may be one of the important mechanisms of concomitant AF in patients with *JAK2* ^*V617F*^‐positive MPN.

To facilitate easy risk stratification of AF, we explored the development of a simple risk prediction model that can easily be used by clinicians. ROC validation showed that the prediction efficiency of this model was high, and there was a significant difference in AF‐free survival between the high‐risk group and the low‐risk group, which was important for the risk stratification for concomitant AF in patients with *JAK2* ^*V617F*^‐positive MPN.

The most important drugs for the treatment of MPN include hydroxyurea, interferon‐α and ruxolitinib. In this study, we explored the associations of different drugs on the occurrence of AF. In our cohort, either interferon‐α or ruxolitinib use was associated with longer AF‐free survival in patients in the high‐risk group. Interferon‐α showed the effects in reducing *V617F*% [30] and immune regulation [31]. Ruxolitinib, a JAK1/*JAK2* inhibitor, improves the number and function of immune cells and inhibit the secretion of proinflammatory factors [32, 33, 34]. Interferon‐α and ruxolitinib may prolong the AF‐free survival in patients of high‐risk group by improving the inflammatory microenvironment and inhibiting the secretion of proinflammatory factors.

### 4.1 Strengths and Limitations

The major strength of this study lies in our comprehensive analysis of clinical factors, cytokines, and genetic findings through NGS, leading to the development of a risk prediction model for AF risk stratification. The limitations include the small sample size (and event rates), which may introduce potential research bias. The retrospective design is also a limitation, as it may lead to study bias; we only demonstrate associations rather than causality. Future studies should adopt a prospective design to better control for confounding variables and establish causal relationships. Additionally, this study is purely clinical and lacks mechanistic exploration. Future research should focus on mechanistic studies to investigate the biological pathways linking *TET2* mutations and increased IL‐1β with AF.

## 5 Conclusion

AF was significantly associated with higher risks of stroke and mortality in patients with *JAK2* ^*V617F*^‐positive MPN. *TET2* mutation and increased IL‐1β were independent risk factors of AF in patients with *JAK2* ^*V617F*^‐positive MPN. Interferon‐α and ruxolitinib were associated with improved AF‐free survival in patients in the high‐risk group.

## Author Contributions

**Guangshuai Teng:** methodology, data curation, validation, visualization, writing – original draft, project administration, formal analysis, funding acquisition, investigation. **Ke Shang:** validation, investigation, formal analysis, visualization. **Yuhui Zhang:** investigation, formal analysis, project administration, data curation. **Yifan Duan:** investigation, validation, formal analysis. **Chenxiao Du:** investigation, formal analysis. **Yan Wang:** investigation, formal analysis. **Yanqi Li:** investigation, formal analysis. **Huiqin Zhang:** investigation, formal analysis. **Lan Peng:** investigation, formal analysis. **Xiaojing Wei:** investigation, methodology. **Gary Tse:** investigation, validation. **Yuan Zhou:** conceptualization, methodology, supervision, resources, writing – review and editing. **Gregory Y. H. Lip:** investigation, resources. **Tong Liu:** conceptualization, methodology, funding acquisition, formal analysis, project administration, resources, supervision, writing – review and editing. **Wei Yang:** investigation, resources, writing – review and editing. **Minghui Duan:** conceptualization, methodology, supervision, formal analysis, project administration, resources, funding acquisition, writing – review and editing. **Jie Bai:** conceptualization, methodology, writing – review and editing, funding acquisition, formal analysis, project administration, resources, supervision.

## Conflicts of Interest

The authors declare no conflicts of interest.

## Supporting information


Data S1:

Data S2:

## Data Availability

All data supporting the findings of this study are available within the article and its Supporting Information S1 and S2 file and are available from the corresponding author upon reasonable request.
