# Supplementary material for: Incidence, Outcomes and Risk Factors for Atrial Fibrillation in Patients With JAK2V617F ‐Positive Myeloproliferative Neoplasms
Source: Cancer Med. 2025 Jul 3;14(13):e71015. doi: 10.1002/cam4.71015 (PMC12224057; doi:10.1002/cam4.71015)

**Supplementary methods**

**Next generation sequencing**

After collection of bone marrow or peripheral blood of the patients into tubes containing ethylene diamine tetraacetic acid (EDTA) as anticoagulant and isolation of mononuclear cells, 1 μg of DNA was extracted for construction of a whole genomic DNA library. The target genome (325 hematological tumor-related genes) was amplified using PCR primers, the target region DNA was enriched, and sequencing was performed using the Ion Torrent sequencing platform, with an average gene coverage rate of 98.1% and an average sequencing depth of 1314 × and more than 20 × for 95% of the target region DNA. Bioinformatic analysis of the sequenced raw data was performed based on authoritative databases such as CCDS, 1000 Genomes, COSMIC and PolyPhen to determine pathogenic gene mutation sites.

**Allele-specific polymerase chain reaction (PCR)**

Detection of *JAK2^V617F^* gene mutation was performed by nested allele-specific PCR. Primers were as follows: P2: 5′‑CCTCAGAACGTTGATGGCA‑3′; P2r: 5′‑ATTGCTTTCCTTTTTCACAAGA‑3′; Pnf: 5′‑AGCATTTGGTTTTAAATTATGGAGTATATG‑3′; Pmr: 5′‑GTTTTACTTACTCTCGTCTCCACAAAA‑3′. The PCR products were assayed by 2.5% agarose gel electrophoresis, then analyzed under ultraviolet light, and the products were 453 bp, 279 bp (mutant *JAK2^V617F^*) and 229 bp (wild type *JAK2^V617F^*). When the DNA sample content was low, the following primers were used for pre-amplification: P1: 5′ ‑GATCTCCATATTCCAGGCTTACACA‑3′ and P1r: 5′ ‑TATTGTTTGGGCATTGTAACCTTCT‑3′, followed by extraction of 1 μl of pre-amplified product for secondary amplification using the nested PCR method described above.

**Real-time Quantitative PCR using Taqman‑MGB probe**

*JAK2^V617F^* mutation load were measured for *JAK2^V617F^*-positive samples in a quantitative manner using a StepOne real-time quantitative PCR system. Upstream primer: 5′ ‑AAGClTITrCTCACAAGCATITIGGTm‑3′, downstream primer: 5′ ‑AGAAAGGCATrAGAAAGCCTGTAGTl‑3′; wild-type *JAK2^V617F^*: Taqman probe VIC‑5′ ‑TCTCCACAGACACATAC‑3′; mutant *JAK2^V617F^*: Taqman probe FAM‑5′ ‑TCCACAGAAACATAC‑3′. A standard curve was drawn by mixing the homozygous *JAK2^V617F^* mutation with PV samples expressing the wild type *JAK2^V617F^* at different ratios; *JAK2^V617F^* mutation load was calculated as: *V617F*

% = mutant *JAK2^V617F^*/(mutant *JAK2^V617F^* + wild-type *JAK2^V617F^* ) × 100%; detection sensitivity was 1%.

**Detection of cytokine levels**

Multiple soluble proteins, including interleukin (IL) ‑ 1β, IL ‑ 2, IL ‑ 4, IL ‑ 5, IL ‑ 6, IL ‑ 8, IL ‑ 10, IL ‑ 12, IL ‑ 17A, IL ‑ 17F, IL ‑ 22, tumor necrosis factor (TNF) ‑ α, TNF ‑ β, and interferon (IFN) ‑ γ, were captured by microspheres of known volume and fluorescence intensity using liquid chip multifactor flow detection technology. The corresponding fluorescence was detected by flow cytometry, and the content of corresponding cytokines of the sample to be tested was obtained based on the fluorescence intensity.

**Supplementary Tables**

**Table S1.** Clinical characteristics of *JAK2^V617F^*-positive MPN patients.

|  | n=439 |
| --- | --- |
| Follow-up time, y media (range) | 6 (1-29) |
| Age, media (range) | 57 (12-87) |
| Age>60, n (%) | 181 (41.2%) |
| Male, n (%) | 225 (51.3%) |
| Diagnosis |  |
| PV, n (%) | 304 (69.2%) |
| ET, n (%) | 41 (9.3%) |
| PostPV/ET MF, n (%) | 65 (14.8%) |
| PMF, n (%) | 29 (6.6%) |
| At the time of diagnosis |  |
| HB, g/L media (range) | 169 (33-261) |
| WBC, ×10^9^/L media (range) | 9.82 (1.34-41.07) |
| Monocytes, ×10^9^/L media (range) | 0.45 (0.11-4.92) |
| Monocytes>0.6×10^9^/L, n (%) | 60 (19.9%) |
| PLT, ×10^9^/L media (range) | 466.5 (34-1749) |
| Abnormal karyotype, n (%) | 24 (9.9%) |
| Reticular fiber ≥ grade 2, n (%) | 94 (25.4%) |
| Splenomegaly, n (%) | 182 (41.7%) |
| *V617F*%>50%, n (%) | 155 (39.7%) |
| Hypertension, n (%) | 77 (23.8%) |
| Hyperlipidemia, n (%) | 67 (24.1%) |
| Diabetes, n (%) | 17 (5.4%) |
| Smoking, n (%) | 43 (11.5%) |
| LVEF, % media (range) | 64 (46-74) |
| BMI, kg/m2 media (range) | 23.3 (16.0-34.2) |
| Concomitant gene mutation |  |
| *DNMT3A*，n (%) | 28 (6.6%) |
| *TET2*，n (%) | 50 (11.8%) |
| *ASXL1*，n (%) | 35 (8.3%) |
| Therapy |  |
| Hydroxyurea, n (%) | 149 (42.9%) |
| Interferon-*α,* n (%) | 315 (76.1%) |
| Ruxolitinib, n (%) | 91 (25.4%) |
| Antiplatelet, n (%) | 217 (67.0%) |
| Anticoagulant, n (%) | 12 (4.9%) |
| Hemorrhage, n (%) | 22 (9.1%) |
| Thrombosis, n (%) | 120 (33.5%) |
| Arterial thrombosis, n (%) | 111 (31.3%) |
| Venous thrombosis, n (%) | 27 (7.6%) |
| AF, n (%) | 29 (6.61%) |
| Heart failure, n (%) | 47 (19.3%) |
| Renal insufficiency, n (%) | 18 (7.8%) |
| Death, n (%) | 40 (9.1%) |

HB, hemoglobin; HCT, hematocrit; PLT, platelet count; WBC, white blood cell; LVEF, left ventricular ejection fraction; BMI, Body mass index; AF, atrial fibrillation; MF, Myelofibrosis.

**Table S2.** Comparison of the proportion of patients with gene mutations and the increased rate of cytokines in patients with *JAK2^V617F^*- positive MPN between AF group and non-AF group.

|  | AF group | Non-AF group | χ^2^ | *p* |
| --- | --- | --- | --- | --- |
| gene |  |  |  |  |
| *V617F*%＞50%, n (%) | 17 (63.0%) | 138 (38%) | 6.531 | 0.014* |
| *DNMT3A* , n (%) | 7 (7.7%) | 24 (6.9%) | 0.025 | 0.699 |
| *TET2*, n (%) | 13 (50%) | 36 (10.3%) | 33.548 | <0.001*** |
| *ASXL1*, n (%) | 3 (11.5%) | 32 (9.2%) | 0.161 | 0.723 |
| *FAT1*, n (%) | 1 (3.8%) | 24 (6.9%) | 0.357 | 1.000 |
| *EP300*, n (%) | 0 | 14 (4.0%) | 1.083 | 0.611 |
| *CREBBP*, n (%) | 1 (3.8%) | 11 (3.2%) | 0.038 | 0.583 |
| *CUX1*, n (%) | 1 (3.8%) | 10 (2.9%) | 0.082 | 0.551 |
| *ARID1A*, n (%) | 0 | 10 (2.9%) | 0.765 | 1.000 |
| *TP53*, n (%) | 2 (7.7%) | 8 (2.3%) | 2.719 | 0.148 |
| *IDH1*, n (%) | 2 (7.7%) | 7 (2.0%) | 3.340 | 0.123 |
| cytokine |  |  |  |  |
| CRP, n (%) | 8 (53.3%) | 33 (26.2%) | 4.789 | 0.029* |
| IL-1β, n (%) | 9 (60%) | 24 (32%) | 4.220 | 0.04* |
| IL-2, n (%) | 0 | 1 (1.3%) | 0.202 | 0.653 |
| IL-4, n (%) | 0 | 12 (16%) | 2.769 | 0.096 |
| IL-5, n (%) | 1 (6.7%) | 5 (6.7%) | ＜0.001 | 1 |
| IL-6, n (%) | 8 (53.3%) | 20 (26.7%) | 4.147 | 0.042* |
| IL-8, n (%) | 10 (66.7%) | 29 (38.7%) | 4.062 | 0.131 |
| IL-10, n (%) | 2 (13.3%) | 21 (28%) | 1.413 | 0.234 |
| IL-12, n (%) | 0 | 1 (1.3%) | 0.202 | 0.653 |
| IL-17A, n (%) | 1 (6.7%) | 17 (22.7%) | 2.000 | 0.157 |
| IL-17F, n (%) | 0 | 2 (2.7%) | 0.409 | 0.522 |
| IL-22, n (%) | 1 (6.7%) | 21 (28%) | 3.080 | 0.079 |
| IFN-γ, n (%) | 1 (6.7%) | 6 (7.7%) | 0.019 | 0.890 |
| TNF-α, n (%) | 2 (13.3%) | 15 (20%) | 0.363 | 0.547 |
| TNF-β, n (%) | 9 (60%) | 15 (20%) | 10.227 | 0.001** |

CRP, C-reactive protein; IL,interleukin; IFN, interferon; TNF, tumor necrosis factor.

* *p* < 0.05

** *p* < 0.01

*** *p* < 0.001

**Table S3.** Comparison of variant allele frequency and the level of cytokines in patients with *JAK2^V617F^*- positive MPN between AF group and non-AF group.

|  | AF group | Non-AF group | *p* |
| --- | --- | --- | --- |
| variant allele frequency |  |  |  |
| *V617F*, % mean±SD | 51.17.44±24.86 | 42.86±26.02 | 0.093 |
| *DNMT3A*, % mean±SD | 3.51±12.52 | 1.64±7.33 | 0.811 |
| *TET2*, % mean±SD | 16.01±20.73 | 3.14±0.78 | <0.001*** |
| *ASXL1*, % mean±SD | 4.29±12.38 | 2.61±10.22 | 0.668 |
| *FAT1*, % mean±SD | 1.91±9.79 | 3.10±11.87 | 0558 |
| *EP300*, % mean±SD | 0 | 1.74±8.74 | 0.299 |
| *CREBBP*, % mean±SD | 2.13±10.85 | 1.40±8.28 | 0.825 |
| *CUX1*, % mean±SD | 1.93±9.83 | 1.40±8.25 | 0.777 |
| *ARID1A*, % mean±SD | 0 | 1.34±7.86 | 0.382 |
| *TP53*, % mean±SD | 3.85±3.67 | 0.55±4.57 | 0.092 |
| *IDH1*, % mean±SD | 3.72±13.15 | 0.68±5.47 | 0.067 |
| levesl of cytokines |  |  |  |
| CRP, mg/dL mean±SD | 5.43±6.75 | 1.36±3.11 | 0.008** |
| IL-1β, pg/mL mean±SD | 5.82±5.63 | 2.70±2.73 | 0.005** |
| IL-2, pg/mL mean±SD | 1.50±1.02 | 1.67±1.89 | 0.905 |
| IL-4, pg/mL mean±SD | 1.75±0.91 | 2.65±4.36 | 0.534 |
| IL-5, pg/mL ±SD | 1.80±1.11 | 1.60±1.21 | 0.401 |
| IL-6, pg/mL mean±SD | 21.50±31.50 | 15.12±34.78 | 0.001** |
| IL-8, pg/mL mean±SD | 202.07±256.65 | 51.05±102.73 | 0.010* |
| IL-10, pg/mL mean±SD | 2.47±1.51 | 4.37±5.71 | 0.414 |
| IL-12, pg/mL mean±SD | 1.80±1.84 | 1.44±1.70 | 0.231 |
| IL-17A, pg/mL mean±SD | 2.13±2.23 | 4.41±6.24 | 0.502 |
| IL-17F, pg/mL mean±SD | 0.93±0.70 | 1.51±3.47 | 0.816 |
| IL-22, pg/mL mean±SD | 1.91±2.05 | 4.72±14.16 | 0.366 |
| IFN-γ, pg/mL mean±SD | 1.72±1.19 | 2.41±3.99 | 0.603 |
| TNF-α, pg/mL mean±SD | 2.40±1.82 | 2.58±2.55 | 0.596 |
| TNF-β, pg/mL mean±SD | 2.29±1.06 | 1.69±1.54 | 0.016* |

CRP, C-reactive protein; IL,interleukin; IFN, interferon; TNF, tumor necrosis factor.

* *p* < 0.05

** *p* < 0.01

*** *p* < 0.001

**Table S4** Univariable and multivariable Cox regression analyses for AF-free survival.

|  | **Univariable analysis** | | **Multivariable analysis** | | |
| --- | --- | --- | --- | --- | --- |
|  | **χ^2^** | ***p*** | **HR** | **95%CI** | ***p*** |
| Age>60 | 13.028 | <0.001*** | 3.010 | 0.391~23.154 | 0.290 |
| gender | 4.406 | 0.110 |  |  |  |
| Reticular fiber ≥ grade 2 | 7.124 | 0.008** | 0.685 | 0.184~2.510 | 0.568 |
| Abnormal karyotype | 0.058 | 0.810 |  |  |  |
| Hypertension | 0.097 | 0.755 |  |  |  |
| Hyperlipidemia | 0.069 | 0.793 |  |  |  |
| Diabetes | 0.274 | 0.600 |  |  |  |
| Smoking | <0.001 | 0.992 |  |  |  |
| Hyperuricemia | 2.457 | 0.117 |  |  |  |
| Antiplatelet | 1.402 | 0.236 |  |  |  |
| Anticoagulant | 0.447 | 0.504 |  |  |  |
| *V617F*%＞50% | 5.453 | 0.020* | 3.946 | 0.888~17.533 | 0.071 |
| *DNMT3A* mutation | 0.558 | 0.455 |  |  |  |
| *TET2* mutation | 20.596 | <0.001*** | 4.361 | 1.053~18.056 | 0.042* |
| *ASXL1* mutation | 0.201 | 0.654 |  |  |  |
| *FAT1* mutation | 0.723 | 0.395 |  |  |  |
| *EP300* mutation | 1.511 | 0.219 |  |  |  |
| *CREBBP* mutation | 0.018 | 0.892 |  |  |  |
| *CUX1* mutation | 0.019 | 0.890 |  |  |  |
| *ARID1A* mutation | 1.759 | 0.185 |  |  |  |
| *TP53* mutation | 1.427 | 0.232 |  |  |  |
| *IDH1* mutation | 2.225 | 0.136 |  |  |  |
| CRP>0.8mg/dL | 4.697 | 0.030* | 1.839 | 0.348~9.726 | 0.474 |
| IL-1β>3.4 pg/mL | 6.128 | 0.013* | 5.476 | 1.547~28.123 | 0.012* |
| IL-2>6.64 pg/mL | 0.125 | 0.724 |  |  |  |
| IL-4>4.19 pg/mL | 2.876 | 0.090 |  |  |  |
| IL-5>4.15 pg/mL | 0.010 | 0.922 |  |  |  |
| IL-6>11.09 pg/mL | 4.893 | 0.027* | 0.578 | 0.095~3.523 | 0.553 |
| IL-8>15.71 pg/mL | 3.010 | 0.222 |  |  |  |
| IL-10>4.5 pg/mL | 0.938 | 0333 |  |  |  |
| IL-12>10.18 pg/mL | 0.125 | 0.724 |  |  |  |
| IL-17A>4.74 pg/mL | 2.217 | 0.137 |  |  |  |
| IL-17F>4.66 pg/mL | 0.616 | 0.432 |  |  |  |
| IL-22>3.64 pg/mL | 3.308 | 0.069 |  |  |  |
| IFN-γ>4.43 pg/mL | 0.001 | 0.969 |  |  |  |
| TNF-α>4.50 pg/mL | 0.512 | 0.474 |  |  |  |
| TNF-β>2.54 pg/mL | 12.760 | <0.001*** | 3.313 | 0.539~20.344 | 0.196 |

CRP, C-reactive protein; IL,interleukin; IFN, interferon; TNF, tumor necrosis factor.

* *p* < 0.05

*** *p* < 0.001

**Supplementary Figures**

**Figure S1.**Observed incidence of AF in *JAK2^V617F^*-positive MPN patients. (A) Clinical patterns of AF. (B) Incidence of AF in PV, ET, Post PV/ET MF, PMF. (C) Cumulative incidence function plot of AF.

**Figure S2.** Kaplan-Meier survival curves showing AF-free survival according to treatment status in the low-risk group. (A) Interferon-α. (B) Ruxolitinib. (C) Hydroxyurea.

**Figure S1**


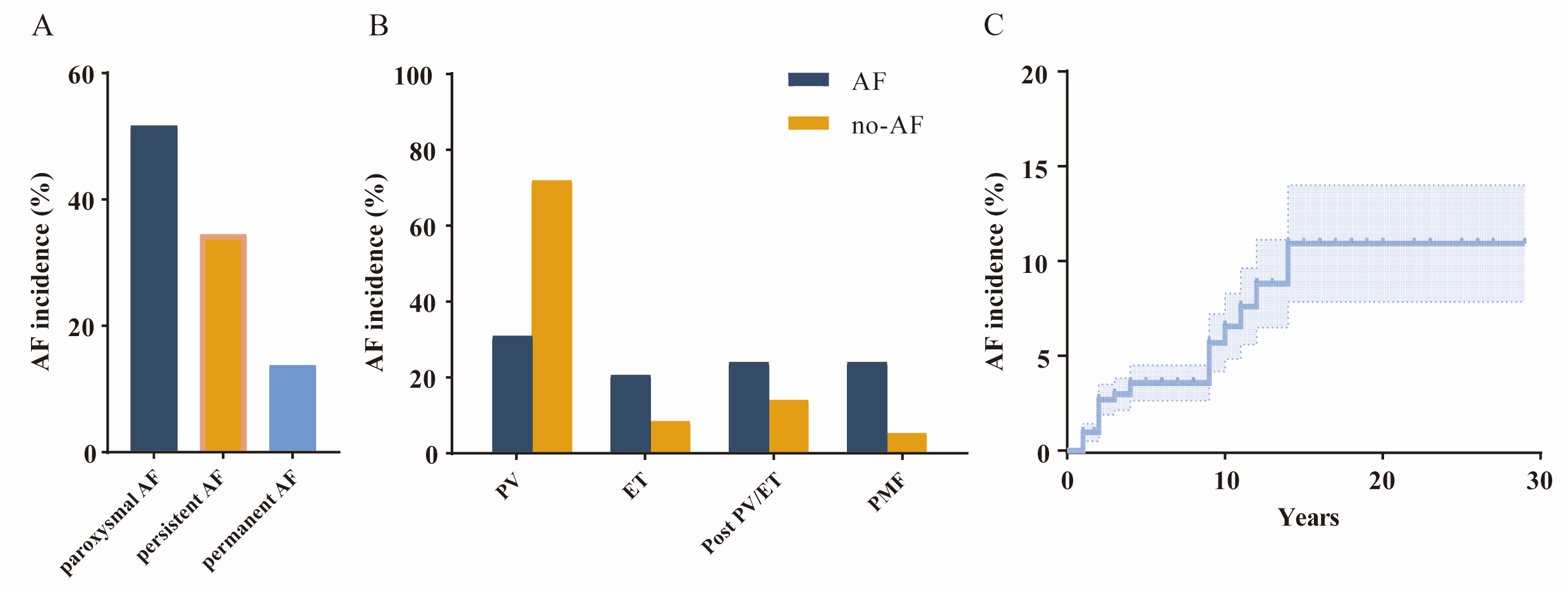


AF, atrial fibrillation; PV, Polycythemia Vera; ET, Essential Thrombocythemia; MF, Myelofibrosis; PMF, Primary Myelofibrosis; * *p* < 0.05.

**Figure S2**


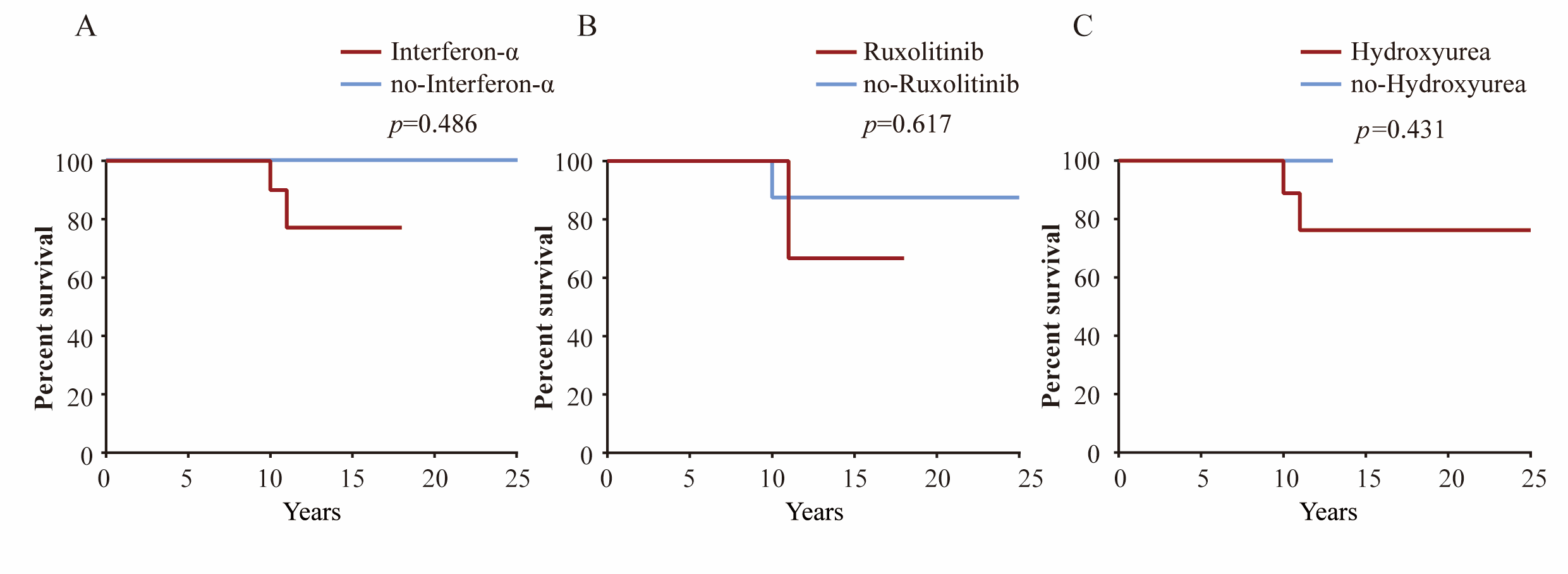

Supplement: Supplementary file 1 — Data S1: [file CAM4-14-e71015-s002.docx]
